# A HAND2 Loss-of-Function Mutation Causes Familial Ventricular Septal Defect and Pulmonary Stenosis

**DOI:** 10.1534/g3.115.026518

**Published:** 2016-02-08

**Authors:** Yu-Min Sun, Jun Wang, Xing-Biao Qiu, Fang Yuan, Ruo-Gu Li, Ying-Jia Xu, Xin-Kai Qu, Hong-Yu Shi, Xu-Min Hou, Ri-Tai Huang, Song Xue, Yi-Qing Yang

**Affiliations:** * Department of Cardiology, Jing’an District Central Hospital, 200040 Shanghai, China; † Department of Cardiology, Shanghai Chest Hospital, Shanghai Jiao Tong University, 200030, China; ‡ Department of Cardiovascular Surgery, Renji Hospital, School of Medicine, Shanghai Jiao Tong University, 200127, China

**Keywords:** congenital heart disease, genetics, transcription factor, HAND2, reporter gene assay

## Abstract

Congenital heart disease (CHD) is the most common developmental abnormality, and is the leading noninfectious cause of mortality in neonates. Increasing evidence demonstrates that genetic defects play an important role in the pathogenesis of CHD. However, CHD exhibits substantial heterogeneity, and the genetic determinants for CHD remain unknown in the overwhelming majority of cases. In the current study, the coding exons and flanking introns of the *HAND2* gene, which encodes a basic helix-loop-helix transcription factor essential for normal cardiovascular development, were sequenced in 192 unrelated patients with CHD, and a novel heterozygous mutation, p.S65I, was identified in a patient with congenital ventricular septal defect (VSD). Genetic analysis of the index patient’s pedigree revealed that the mutation was present in all seven affected family members available, but absent in the 13 unaffected family members examined. Besides, in addition to VSD, five of the proband’s close relatives also had pulmonary stenosis (PS), and the proband’s son also had double outlet right ventricle (DORV). The missense mutation, which altered an evolutionarily conserved amino acid, was absent in 300 unrelated, ethnically matched healthy individuals. Biological analyses using a dual-luciferase reporter assay system showed that the mutant HAND2 was associated with significantly diminished transcriptional activity. Furthermore, the mutation abolished the synergistic activation between HAND2 and GATA4, as well as NKX2.5—two other cardiac core transcriptional factors that have been causally linked to CHD. These findings indicate that HAND2 loss-of-function mutation contributes to human CHD, perhaps via its interaction with GATA4 and NKX2.5.

Congenital heart disease (CHD) is the most prevalent birth defect in humans, with an estimated prevalence of 1% in newborns (Hoffman and Kaplan 2002). Severe CHD may contribute to impaired exercise performance, poorer health-related quality of life, delayed fetal brain development, thromboembolism, pulmonary hypertension, cardiac arrhythmias, congestive heart failure, and sudden cardiac death (Mozaffarian *et al.* 2015). Consequently, CHD has led to substantial cardiovascular morbidity and mortality, conferring a heavy socioeconomic burden on patients’ families and social health care systems (Mozaffarian *et al.* 2015). Despite the important clinical significance, the etiologies responsible for CHD are largely undefined.

In vertebrates, cardiac morphogenesis is a complex and dynamic process that requires an accurate spatiotemporal cooperation of multiple transcription factors, adhesion molecules, ion channels, signaling molecules, and structural proteins, and both environmental and genetic causes may disrupt this biological process of cardiogenesis, giving rise to a wide spectrum of CHD (Srivastava and Olson 2000). Although environmental risk factors for CHD are also referred to, a growing number of studies strongly suggest genetic defects as the predominant cause of CHD. Currently, mutations in more than 60 genes have been related to CHD (Al Turki *et al.* 2014; Andersen *et al.* 2014; Ta-Shma *et al.* 2014; Werner *et al.* 2014; Ramachandran *et al.* 2015). Among these well-established CHD-causing genes, those coding for cardiac transcription factors are the most frequently associated with CHD, highlighting the crucial roles of cardiac transcription factors in cardiovascular development and disease (McCulley and Black 2012).

As the only two members of the basic helix-loop-helix family of transcription factors, both HAND1 and HAND2 have been demonstrated to play critical roles in normal cardiovascular development, and mice with deletions in either *Hand1* and *Hand2* mice show cardiovascular developmental malformations (Vincentz *et al.* 2011). Moreover, mutations in human *HAND1* that result in gain or loss of protein function have been causally linked to a wide array of CHD (Reamon-Buettner *et al.* 2008, 2009; Cheng *et al.* 2012). Given that the expression profiles and functional characteristics of *HAND2* overlap at least partially with those of *HAND1* (Srivastava *et al.* 1995, 1997; McFadden *et al.* 2005; Vincentz *et al.* 2011), screening *HAND2* as a prime candidate gene in CHD patients is justifiable (Shen *et al.* 2010; Töpf *et al.* 2014).

## Materials and Methods

### Study participants

In this study, 192 unrelated patients with CHD were recruited from the Han Chinese population. The study also included 33 relatives of the index patient who harbored an identified *HAND2* mutation. A total of 300 ethnically matched, unrelated individuals without CHD confirmed by echocardiography were enlisted as controls. Detailed clinical assessment of the study participants was made, which included review of medical records, anthropometric measurement, physical examination for malformation, two-dimensional echocardiography, standard 12-lead electrocardiogram, and chest X-ray radiography. Various types of CHD were diagnosed by two-dimensional and Doppler echocardiography. When necessary, transesophageal echocardiography, cardiac catheterization, angiography, and intervention were carried out for further clarification of the anatomic structures. Nonsyndromic CHD cases only were included. To exclude DiGeorge syndrome, proband samples were screened for chromosome 22q11.2 deletion as previously described (Töpf *et al.* 2014). However, a microarray was not done to exclude other copy-number variants (Soemedi *et al.* 2012). The study complies with the ethical guidelines of the 1975 Declaration of Helsinki. The research protocol was approved by the local medical research ethics committee. Written informed consent was obtained from the study participants or their guardians before commencement of the investigation.

### Genetic scan of HAND2 for mutation

Peripheral venous blood specimens were drawn from the study subjects. Genomic DNA was isolated from blood leukocytes using the Wizard Genomic DNA Purification Kit (Promega, Madison, WI). The genomic DNA sequence of the human *HAND2* gene (accession no. NC_000004) was obtained from the GenBank database (http://www.ncbi.nlm.nih.gov/genbank/). Using the online Primer 3 program (http://primer3.ut.ee/), primers to amplify the coding exons and splicing boundaries of *HAND2* by polymerase chain reaction (PCR) were designed as shown in Table 1. PCR was performed with HotStar Taq DNA Polymerase (Qiagen, Hilden, Germany) on a Veriti Thermal Cycler (Applied Biosystems, Foster, CA). The amplicons were sequenced using a BigDye Terminator v3.1 Cycle Sequencing Kit (Applied Biosystems), under an ABI PRISM 3130 XL DNA Analyzer (Applied Biosystems). For an identified sequence variation, the Single Nucleotide Polymorphism (SNP; http://www.ncbi.nlm.nicch.gov/SNP), the 1000 Genomes Project (1000 GP; http://www.1000genomes.org), and the Exome Variant Server (EVS; http://evs.gs.washington.edu/EVS) databases were consulted to verify its novelty.

**Table 1:** Primers used to amplify the coding exons, and exon–intron boundaries of the *HAND2* gene

Coding Exon	Forward Primer (5′ to 3′)	Reverse Primer (5′ to 3′)	Size (bp)
1-a	CGAGAGGATTCTGCCTCCGC	ACAGGGCCATGCTGTAGTCG	550
1-b	GGTAGGTGGTTTTCCCCACCA	GCCCAATTGGAAAGAGGCCG	624
2	GGTTCACTGTCTCCTCCGGC	CGGGATCCCTTACCACACGG	483

### Multiple sequence alignments of HAND2 proteins across species

The amino acid sequences of the HAND2 proteins from various species were aligned by Clustal W (http://www.clustal.org/clustal2).

### Prediction of the causative potential of HAND2 sequence variation

The disease-causing potential of *HAND2* sequence variation was predicted by MutationTaster (http://www.mutationtaster.org), PolyPhen-2 (http://genetics.bwh.harvard.edu/pph2), and PROVEN (http://provean.jcvi.org/index.php).

### Expression vectors and site-targeted mutagenesis

Full-length *HAND2* cDNAs were amplified from the templates of human heart cDNAs prepared as described previously (Shi *et al.* 2014), digested with *Eco*RI and *Not*I, and then inserted into the *Eco*RI–*Not*I sites of the pcDNA3.1 vector (Invitrogen, Carlsbad, CA). The mutation was introduced into the wild-type HAND2-pcDNA3.1 by site-directed mutagenesis with a complementary pair of primers containing the identified DNA base substitution using the QuickChange II XL Site-Directed Mutagenesis Kit (Stratagene, La Jolla, CA). The mutant HAND2-pcDNA3.1 was confirmed by sequencing. The expression vectors NKX2.5-pEFSA and GATA4-pSSRa, as well as the ANF-luciferase reporter (ANF-luc) plasmid, which contains 2600 bp upstream of the transcriptional start site of the *ANF* gene and expresses firefly luciferase, were generous gifts from Dr. Ichiro Shiojima at Chiba University School of Medicine, Japan.

### Cell culture, transfection, and luciferase assays

Human embryonic kidney HEK293 cells and human epithelial cervical cancer HeLa cells were maintained in Dulbecco’s modified Eagle’s medium containing 10% fetal bovine serum (Life Technologies, Grand Island, NY). Cells at approximately 90% confluence were transfected with plasmids using lipofectamine 2000 reagent (Invitrogen). As an internal control, the vector pGL4.75 (Promega), which expresses the Renilla luciferase, was used to normalize transfection efficiency. In transfection of HEK293 cells, 0.8 μg of wild-type HAND2-pcDNA3.1, 0.8 μg of mutant HAND2-pcDNA3.1, 0.4 μg of wild-type HAND2-pcDNA3.1, or 0.4 μg of wild-type HAND2-pcDNA3.1 in combination with 0.4 μg of mutant HAND2-pcDNA3.1 was used in the presence of 1.0 μg of ANF-luc and 0.04 μg of pGL4.75. In transfection of HeLa cells, the same amount (0.6 μg) of plasmid DNA (wild-type HAND2-pcDNA3.1, mutant HAND1-pcDNA3.1, GATA4-pSSRa or NKX2.5-pEFSA) was used alone or together, in the presence of 1.0 μg of ANF-luc and 0.04 μg of pGL4.75. Cells were harvested and lysed 36 h after transfection, and then the firefly and Renilla luciferase activities were measured with the Dual-Glo luciferase assay system (Promega). All transfection experiments were conducted in triplicate, and repeated at least three times independently. Results were shown as fold activation of firefly luciferase relative to Renilla luciferase.

### Statistical analysis

Statistical analyses were performed using the Statistical Package for Social Sciences for Windows (SPSS, Chicago, IL). Numerical variables were expressed as mean ± SD, and categorical variables were noted as numbers and percentages. Continuous variables were compared between two groups with the unpaired Student’s *t*-test when normally distributed, or the Mann-Whitney test otherwise, and for the comparison of categorical variables, the χ^2^ test or Fisher’s exact test was used, as indicated. Two-tailed p-values below 0.05 were considered statistically significant.

### Data availability

The authors state that all data necessary for confirming the conclusions presented in the article are represented fully within the article.

## Results

### Clinical evaluation of the study subjects

In contrast to 300 unrelated nonCHD control subjects, 192 unrelated patients with CHD underwent comprehensive clinical evaluation, including validation of the CHD by echocardiogram or further by cardiac surgery. Of the 192 CHD patients, 28 had a positive family history. Based on reviews of medical histories, and analyses of medical records encompassing echocardiographic images, the control persons had neither CHD nor positive family history of CHD. The demographic profiles and baseline clinical features of the CHD patients are given in Table 2.

**Table 2:** Demographic profiles and baseline clinical characteristics of the study subjects with congenital heart disease (*n* = 192)

Variables	Statistics
Age (years)	8.62 ± 10.47
Male (%)	93 (48)
Female (%)	99 (52)
Positive family history of congenital heart disease (%)	28 (15)
Distribution of different kinds of congenital heart diseases	125 (65)
Isolated congenital heart disease (%)	47 (24)
Ventricular septal defect (%)	31 (16)
Atrial septal defect (%)	18 (9)
Patent ductus arteriosus (%)	7 (4)
Aortic stenosis (%)	5 (3)
Pulmonary stenosis (%)	5 (3)
Coarctation of the aorta (%)	12 (6)
Other isolated congenital heart disease (%)	67 (35)
Complex congenital heart disease (%)	28 (15)
Tetralogy of Fallot (%)	12 (6)
Atrial septal defect + ventricular septal defect (%)	6 (3)
Ventricular septal defect + double outlet of right ventricle (%)	6 (3)
Ventricular septal defect + transposition of the great arteries (%)	5 (3)
Ventricular septal defect + truncus arteriosus (%)	10 (5)
Other complex congenital heart disease (%)	111 (58)
Treatment	75 (39)
Cardiac surgery (%)	6 (3)
Catheter-based repair (%)	
Follow-up (%)	

Data are expressed as mean ± SD, number, or percentage.

### Identification of a novel HAND2 mutation

**Figure 1:**
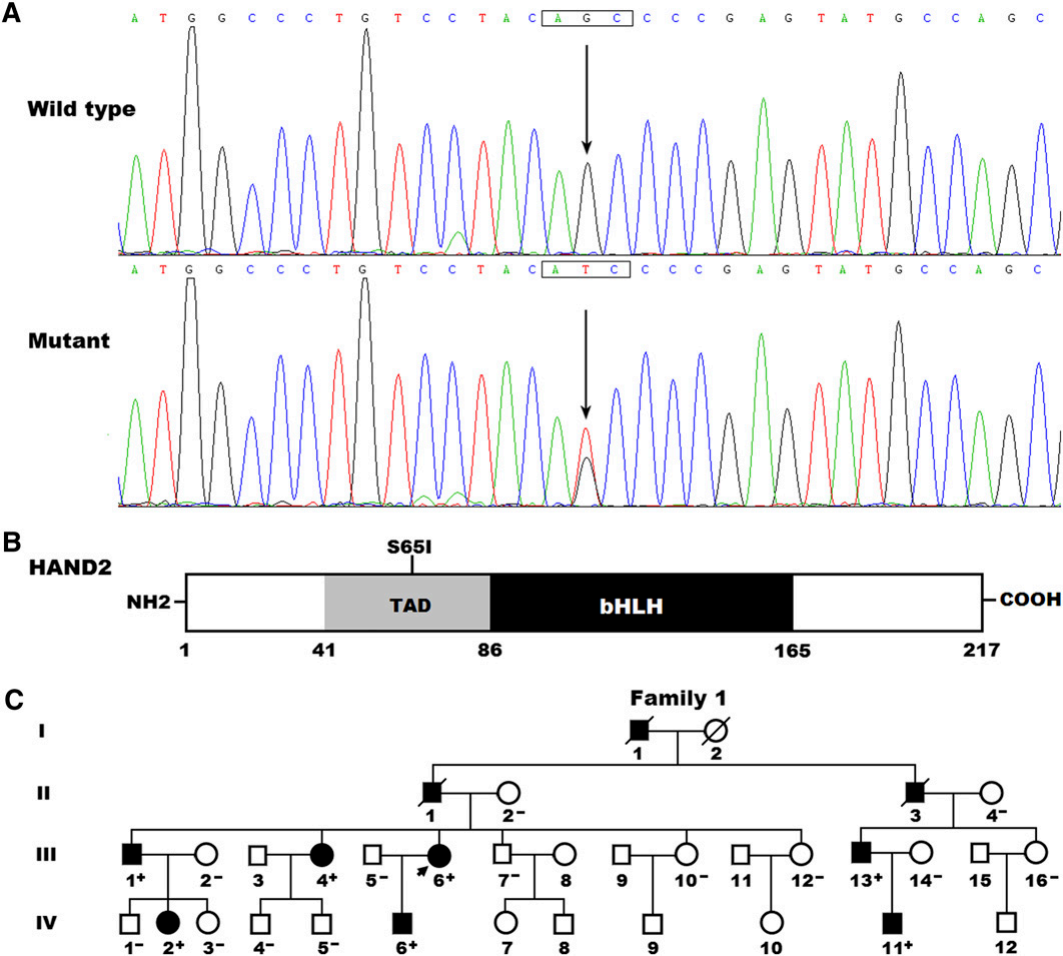
A novel HAND2 mutation associated with congenital heart disease. (A) Sequence electropherograms showing the heterozygous *HAND2* mutation as well as its wild-type control. The arrow indicates the heterozygous nucleotides of G/T in the proband (mutant), or the homozygous nucleotides of G/G in a control individual (wild type). The rectangle marks the nucleotides comprising a codon of *HAND2*. (B) A schematic diagram depicting the structural domains of the HAND2 protein. The mutation detected in a family with congenital heart disease (CHD) is marked above the structural domains. NH2, amino-terminus; TAD, transcriptional activation domain; bHLH, basic helix-loop-helix; COOH, carboxyl-terminus. (C) A family with CHD and a HAND2 mutation. The family was designated as family 1. Family members are identified by generations and numbers. Square, male family member; circle, female member; symbol with a slash, deceased member; closed symbol, affected member; open symbol, unaffected member; arrow, proband; “+”, carrier of the mutation; and “–”, noncarrier.

By sequence analysis of the *HAND2* gene in 192 unrelated patients with CHD, a substitution of thymine for guanine in the second nucleotide of codon 65 (c.194G > T), predicting the transition of serine to isoleucine at amino acid position 65 (p.S65I), was detected in a patient with congenital subaortic ventricular septal defect (VSD) and a positive family history of CHD. The detection rate of HAND2 mutation in this cohort of CHD cases was about 0.52%, which was higher in familial (∼3.57%) than in sporadic (0%) cases. The DNA sequencing electropherograms showing the heterozygous *HAND2* mutation of c.194G > T in contrast to its control sequence are displayed in Figure 1A. A schematic drawing of HAND2 depicting the functionally important structural domains, and the location of the mutation detected in this study, is presented in Figure 1B. The missense mutation, which was absent in the 300 control individuals, was not found in the SNP, 1000 GP and EVS databases (accessed again on October 6, 2015). Genetic analysis of the index patient’s available relatives revealed that the mutation was present in all affected living family members, but absent in unaffected family members examined. Analysis of the pedigree unveiled that the mutation cosegregated with VSD, which was transmitted as an autosomal dominant trait in the family with complete penetrance. In addition to VSD, five of the proband’s relatives (I-1, II-2, III-1, III-13, and IV-11) also suffered from pulmonary stenosis (PS), and the proband’s son (IV-6) also had double outlet right ventricle (DORV). The pedigree structure of the family is shown in Figure 1C. The phenotypic characteristics and status of HAND2 mutation of affected family members are summarized in Table 3.

**Table 3:** Phenotypic characteristics and status of HAND2 mutation of the pedigree members affected with congenital heart disease

Subject Information			Phenotype	Genotype
Identity	Gender	Age (Years)	Cardiac Structural Defects	HAND2 Mutation
Family 1				S65I
I-1	M	48*^a^*	VSD, PS	NA
II-1	M	60*^a^*	VSD	NA
II-1I	M	56*^a^*	VSD, PS	NA
III-1	M	38	VSD, PS	+/–
III-4	F	36	VSD	+/–
III-6	F	32	VSD	+/–
III-13	M	34	VSD, PS	+/–
IV-2	F	9	VSD	+/–
IV-6	M	4	VSD, DORV	+/–
IV-11	M	7	VSD, PS	+/–

M, male; F, female; VSD, ventricular septal defect; PS, pulmonary stenosis; DORV, double outlet of right ventricle; NA, not available; +/–, heterozygote.

a Age at death.

### Conservation of mutated amino acid residue in the HAND2 protein

**Figure 2:**
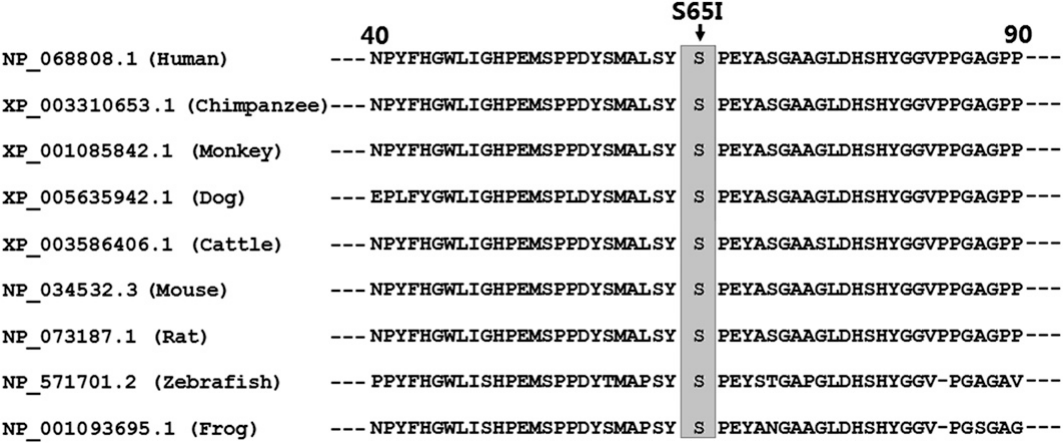
Alignment of multiple HAND2 proteins across species. Alignment of the HAND2 proteins from various species showed that the altered serine at amino acid 65 of human HAND2 was completely conserved evolutionarily.

Alignment of multiple HAND2 protein sequences from various species showed that the altered serine at amino acid 65 of human HAND2 was completely conserved evolutionarily (Figure 2).

### Causative potential of the HAND2 sequence variation

The variation c.194G > T of *HAND2* was predicted to be disease-causing by MutationTaster, with a p-value of about 1.000. The amino acid substitution p.S65I of HAND2 was also predicted to be possibly damaging by PolyPhen-2, with a score of 0.938 (sensitivity: 0.80; specificity: 0.94), and deleterious by PROVEN, with a PROVEN score of –2.663.

### Effect of mutant HAND2 on the transcriptional activation of the ANF promoter

**Figure 3:**
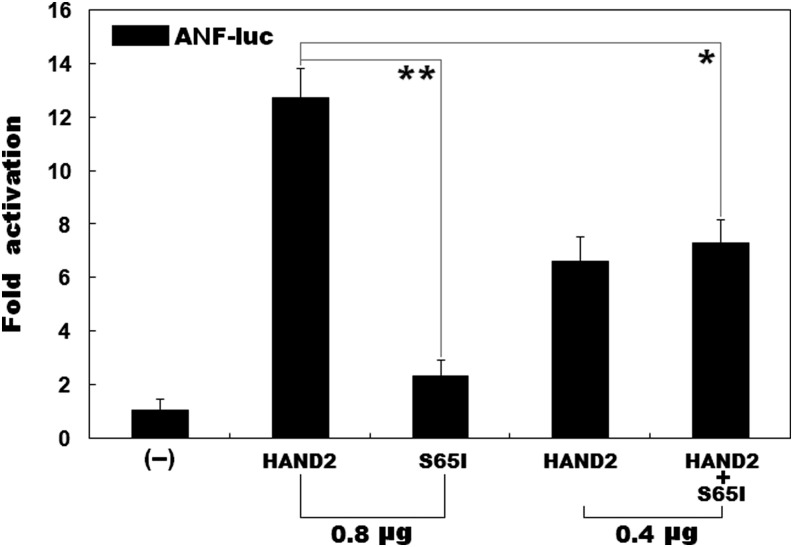
Functional impairment of mutated HAND2 in activation of a target gene. A dual-luciferase reporter assay was used to evaluate the functional consequence of the HAND2 mutation in a cellular context of HEK293 cells, showing that the mutant HAND2 was associated with significantly diminished transcriptional activation of the *ANF* promoter. Three independent experiments were carried out in triplicate, with mean and SD shown. ** denotes *t* = 14.3905, p = 0.0001; * indicates *t* = 6.6623, p = 0.0026, when compared with wild-type HAND2.

As shown in Figure 3, the same amount (0.8 μg) of wild-type and S65I-mutant HAND2 transactivated the *ANF* promoter by ∼13-fold and ∼2-fold, respectively. When 0.4 μg of wild-type HAND2 was used alone, or together with 0.4 μg of S65I-mutant HAND2, the induced activation of the *ANF* promoter was equal to ∼7-fold.

### Effect of the mutation on the synergistic activation of the ANF promoter between HAND2 and GATA4 or NKX2.5

**Figure 4:**
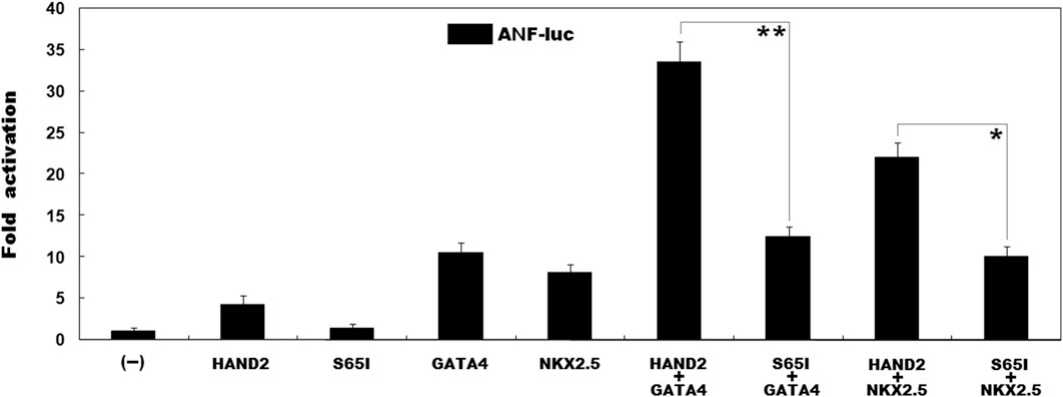
Impaired synergistic activation between HAND2 and GATA4 as well as NKX2.5 due to the mutation. The functional effect of the mutant HAND2 protein on cooperative activation of the *ANF* promoter with GATA4 or NKX2.5 was tested in HeLa cells, showing that the mutant HAND2 had significantly decreased activation of the reporter in synergy with GATA4 as well as NKX2.5. Experiments were conducted in triplicate, with mean and SDs shown. ** indicates *t* = 13.4046, p = 0.0002; * indicates *t* = 9.3988, p = 0.0007, when compared with wild-type counterparts.

As shown in Figure 4, the same amount (0.6 μg) of wild-type and S65I-mutant HAND2 activated the *ANF* promoter by ∼4-fold and ∼1-fold, respectively. In the presence of 0.6 μg of wild-type *GATA4*, the same amount (0.6 μg) of wild-type and S65I-mutant HAND2 activated the *ANF* promoter by ∼34-fold and ∼12-fold, respectively, while in the presence of 0.6 μg of wild-type *NKX2.5*, the same amount (0.6 μg) of wild-type and S65I-mutant HAND2 activated the *ANF* promoter by ∼22-fold and ∼10-fold, respectively.

## Discussion

In the present study, a novel heterozygous mutation of p.S65I in HAND2 was discovered in a family with CHD, including VSD, PS and DORV. The mutation cosegregated with CHD, which was transmitted in an autosomal dominant mode in the family with complete penetrance. The mutation, which was absent in the 600 reference chromosomes, and altered an amino acid conserved evolutionally across species, was predicted to be pathogenic. Biological analyses revealed that the S65I-mutant HAND2 had a significant diminished transcriptional activity. Furthermore, the S65I mutation markedly reduced the synergistic activation between HAND2 and GATA4 as well as NKX2.5. Hence, it is very likely that mutated *HAND2* contributes to the pathogenesis of CHD in these mutation carriers. Given that VSD is the most common type of CHD, this study highlights the crucial role of HAND2 in regulation of septation during cardiac morphogenesis, with potential implications for medical care and genetic counseling in this large population.

Human *HAND2* maps on chromosome 4q33, and encodes a protein of 217 amino acids. The HAND2 protein has two functionally important structural domains: a transcriptional activation domain (TAD), and a basic helix-loop-helix (bHLH) domain. TAD is responsible for the transcriptional activation of target genes, while bHLH is required for DNA binding and protein–protein interactions (Dai and Cserjesi 2002). Previous experiments substantiated that HAND2 transactivated multiple target genes, including *ANF*, expressed during embryonic cardiogenesisalone, or in synergy with cooperative partners such as GATA4, NKX2.5, and MEF2C (Thattaliyath *et al.* 2002; Zang *et al.* 2004). In this study, the HAND2 mutation identified in CHD patients is located in TAD, and reporter gene assays showed that mutant HAND2 had significantly decreased transcriptional activation of the *ANF* promoter. Furthermore, the mutation markedly reduced the synergistic activation between HAND2 and GATA4 as well as NKX2.5—the two other cardiac core transcription factors most commonly associated with CHD in humans (McCulley and Black 2012; Andersen *et al.* 2014). These findings suggest that *HAND2* haploinsufficiency is potentially an alternative molecular mechanism of CHD.

Previous studies in animal models have demonstrated that HAND2 is crucial for cardiovascular morphogenesis. In zebrafish, *HAND* was expressed in the heart, and *Hand2*-mutant embryos had myocardial developmental defects from early stage, including a dramatically reduced number of myocardial precursors, and an improperly patterned myocardial tissue (Yelon *et al.* 2000). In chicks, HAND2 was expressed in the bilateral heart primordia, and throughout the primitive tubular heart, as well as its derivatives during embryonic development, and incubation of stage 8 chick embryos with *Hand2* and *Hand1* antisense oligonucleotides revealed that either oligonucleotide alone had no effect on embryogenesis, whereas together they arrested cardiac development at the looping heart tube stage (Srivastava *et al.* 1995). In mice, HAND2 was expressed in mesodermal and neural crest-derived structures of the developing heart, and deletion of *Hand2* in mouse embryos led to embryonic lethality from right ventricular hypoplasia and vascular defects (Srivastava *et al.* 1997). In mouse embryos rescued by activating adrenergic receptors, loss of HAND2 in the cardiac neural crest lineage caused misalignment of the outflow tract and aortic arch arteries, giving rise to PS, interrupted aortic artery, retroesophageal right subclavian artery, DORV, and VSD (Morikawa and Cserjesi 2008). In addition, conditional knockout of *Hand2* at later stages of development, and in restricted domains of the second heart field, resulted in a spectrum of cardiac anomalies including VSD (Tsuchihashi *et al.* 2011), while endocardial ablation of *Hand2* caused failure to develop a patent tricuspid valve, intraventricular septum defects, and hypotrabeculated ventricles (VanDusen *et al.* 2014). Taken together, these results indicate that genetically compromised HAND2 confers an increased vulnerability to CHD.

In humans, HAND2 was strongly expressed in the heart, and patients with genomic deletions or duplications that involved chromosome 4q33, the locus of *HAND2*, were susceptible to CHD, including VSD, tetralogy of Fallot (TOF), pulmonary atresia, and coarctation of the aorta (Russell *et al.* 1998). Moreover, HAND2 mutations were also found in patients with TOF (Shen *et al.* 2010; Töpf *et al.* 2014). Shen and colleagues (Shen *et al.* 2010) sequenced the *HAND2* gene in 131 patients with congenital defects of the right ventricle, outflow tract, aortic artery, or endocardial cushion, and identified three heterozygous nonsynonymous mutations (p.P11R, p.S36N, and p.V83L) in four patients with TOF. However, the functional effects of these CHD-associated mutations remained unclear. Töpf and coworkers (Töpf *et al.* 2014) sequenced 12 transcription factor genes implicated in cardiac outflow tract development, including *HAND2*, in 93 nonsyndromic cases with TOF, and identified a heterozygous insertion mutation in HAND2, p.A25_A26insAA. Reporter assays showed the mutant HAND protein had significantly decreased transcriptional activation. In summary, these results, along with those of the current study, imply that mutated *HAND2* may be an uncommon cause of CHD in humans.

In conclusion, this study is the first to associate HAND2 loss-of-function mutation with enhanced susceptibility to familial VSD and PS, as well as sporadic DORV, in humans, providing significant insight into the molecular mechanisms of CHD.
